# Exposure to Perfluoroalkyl Chemicals and Cardiovascular Disease: Experimental and Epidemiological Evidence

**DOI:** 10.3389/fendo.2021.706352

**Published:** 2021-07-09

**Authors:** Alessandra Meneguzzi, Cristiano Fava, Marco Castelli, Pietro Minuz

**Affiliations:** Department of Medicine, University of Verona, Verona, Italy

**Keywords:** perfluoroalkyl substances, cardiovascular disease, obesity, diabetes mellitus, arterial hypertension, platelets, thrombosis

## Abstract

Polyfluoro- and perfluoro–alkyl substances (PFAS) are organic chemicals extensively used worldwide for industry and consumer products. Due to their chemical stability, PFAS represent a major cause of environmental pollution. PFAS accumulate in animal and human blood and tissues exerting their toxicity. We performed a review of the epidemiological studies exploring the relationship between exposure to PFAS and thromboembolic cardiovascular disease. An increase in cardiovascular disease or death related to PFAS exposure has been reported from cross-sectional and longitudinal observational studies with evidence concerning the relation with early vascular lesions and atherosclerosis. Several studies indicate an alteration in lipid and glucose metabolism disorders and increased blood pressure as a possible link with cardiovascular thromboembolic events. We also examined the recent evidence indicating that legacy and new PFAS can be incorporated in platelet cell membranes giving a solid rationale to the observed increase risk of cardiovascular events in the populations exposed to PFAS by directly promoting thrombus formation. Exposure to PFAS has been related to altered plasma membrane fluidity and associated with altered calcium signal and increased platelet response to agonists, both *in vitro* and *ex vivo* in subjects exposed to PFAS. All the functional responses are increased in platelets by incorporation of PFAS: adhesion, aggregation, microvesicles release and experimental thrombus formation. These findings offer mechanistic support the hypothesis that platelet-centred mechanisms may be implicated in the increase in cardiovascular events observed in populations chronically exposed to PFAS.

## Introduction

Polyfluoro- and perfluoro–alkyl substances (PFAS) represent a large class of organic chemicals derived from the substitution of hydrogen with fluorine attached to hydrocarbon chain, that has been extensively used since the 1940s (1). Non-polymeric and polymeric PFAS are components of a wide range of industry and consumer products. Due to their unique physical and chemical characteristics, PFAS are used as oil and water repellents and flame retardants, raw materials for fire-fighting equipment and mist suppression as well as coating for cookware, carpets, textiles, component of paints, lubricants and fire-fighting foams (1–4).

Thus, their presence in the environment increased steadily until early 2000 when, at least in some countries, regulatory laws led to a decrease in the industrial utilization, at least of some PFAS (legacy PFAS). Since then, different PFAS, that are considered to accumulate in lower amounts and for shorter periods of time, have been introduced on the market and are currently in use. However, these new formulations are widely used without proper knowledge of their environmental disposition and potential toxicity (1, 5, 6).

The most widely used PFAS have also been extensively studied. Fluorinated substances (more than a thousand chemical compounds exists) are either acids or sulfonate: perfluorodecanoic acid (PFDA), perfluoroheptane sulfonate (PFHpS), perfluorohexane sulfonate (PFHxS), perfluorononanoic acid (PFNA), perfluorooctanoic acid (PFOA), perfluorooctane sulfonate (PFOS), 2-(N-ethyl-perfluorooctane sulfonamido) acetate (EPAH), 2-(N-methyl-perfluorooctane sulfonamido) acetate (MPAH), perfluorobutane sulfonate (PFBS), perfluoroheptanoic acid (PFHP), perfluorooctane sulfonamide (PFSA), perfluoroundecanoic acid (PFUA) and perfluorododecanoic acid (PFDO).

Many PFAS are highly resistant to degradation and represent persistent organic pollutants. Because of the huge number of applications and worldwide distribution of polluting sources, PFAS have been found even in the Arctic region (7). Due to their long half-life, PFAS can accumulate in the blood and tissues of living organisms through the food chain. The chemical structure of individual PFAS, direct environmental exposure, lifestyle, gender and possibly genetic factors play a role in their uptake and retention in humans. Their accumulation in blood and tissues is especially high in people living in industrialised countries near sites of PFAS production where drinking water is contaminated (8, 9). PFAS, and in particular PFOS, PFOA, PFHxS, and PFNA have been detected in the blood of nearly 99% of the U.S. Population examined in National Health and Nutritional Examination Survey (NHANES) 2003–2004 and NHANES 1999–2000 (8). However, several of these compounds have been found circulating in blood at the high ng/mL range of concentrations in heavily exposed subjects (10, 11). Due to tissue accumulation and the kinetics profile of PFOA, the estimated time required for its elimination from plasma in humans is 5 to 8.5 years (12).

PFAS accumulating in human tissues have been shown to be toxic. Research in this field is expanding fast and the spectrum of PFAS biological effects, almost adverse, is being defined (1). In fact, PFAS absorbed by the intestine or inhaled may exert their toxicity acting as “endocrine disruptors” affecting the reproductive system, the function of the thyroid gland, bone metabolism and leading to a number of deleterious health consequences on the immune, the nervous and, possibly, the cardiovascular system (1, 13, 14). In recent years, several observational studied highlighted the relationship between exposure to PFAS and cardiovascular disease and death (15). This information derived either from population-based registries or from longitudinal observational studies comparing the level of exposure with clinical events and control groups. When the clinical outcome was analysed, excess in arterial thrombosis (e.g. myocardial infarction) was identified in most studies. Two possible explanations may be advocated: worsening in cardiovascular risk or a direct prothrombotic activity of PFAS in exposed subjects. The effects of PFAS on cardiovascular risk factors, namely hyperlipidaemia, diabetes mellitus and arterial hypertension, have been extensively investigated. Recently, the mechanisms of PFAS accumulation in human cells are being disclosed. Platelets have been found to accumulate PFAS and to be altered in their function as a consequence of that. We performed a review focused on the epidemiology studies in which the hypothesis that exposure to PFAS is associated with increased risk of cardiovascular events or alteration in cardiovascular structure and function has been investigated. We also analysed the recent scientific evidence linking exposure to legacy and new PFAS to altered platelet function, representing a plausible explanation for an excess of arterial thrombosis in exposed populations.

## Methods for Study Selection

The aim of the present review was to give an overview of the most relevant epidemiological studies dealing with the connection between PFAS and cardiovascular diseases and risk factors. In the second part, the review focuses on the mechanistic studies on PFAS and thrombosis.

The choice of the epidemiological studies to be included in the main text and the tables was based on the sample size, the study design, preferring the longitudinal ones, and the intensity of the exposure.

## PFAS, Cardiovascular Damage and Cardiovascular Events: Epidemiological Evidence

We considered the epidemiological studies concerning the possible association between exposure to PFAS and cardiovascular diseases and cardiovascular risk factors (**Table 1**).

**Table 1:** Clinical and epidemiological studies exploring the rule of PFAS in cardiovascular disease and cardiovascular risk factors.

Author, year and reference	Study Design	Population	n. of subjects	PFAS plasma concentration (ng/mL)	Observation period	Main results
Shankar et al., 2012 (16)	Cohort	US adults ≥ 40 years old from “National Health and Nutrition Examination Survey” (NHANES)	1216	Values in the 4th quartile **PFOA**: ≥ 5.6 for women and ≥ 6.1 for men	4 years	Higher PFOA levels positively associated with cardiovascular disease and peripheral arterial disease
Huang et al., 2018 (2)	Cohort longitudinal	US participants from the NHANES 1999–2014	10859	range in quartile (Qn) Q1:<12.11, Q2: 12.11–20.61, Q3: 20.61–33.6, Q4: > 33.63. Analysed compounds: **PFOA, PFOS, PFHxS, EPAH, MPAH, PFDA, PFBS, PFHP, PFNA, PFSA, PFUA, and PFDO**	approximately 15 years	Total PFAS were positively associated with cardiovascular disease. Serum levels of MPAH and PFDO were positively associated with congestive heart failure; PFNA, PFDA, and PFUA were positively associated with coronary heart disease; PFUA and PFDO were positively associated with angina pectoris; and PFNA was positively associated with heart attack.
Mastrantonio et al., 2018 (17)	Ecological mortality study	Populations from Veneto municipalities with PFAS contaminated and uncontaminated drinking water	41841 deaths	**PFOS**: ≥30, **PFOA**: ≥ 500, **PFUnA**: ≥ 500	33 years	Higher mortality levels for some causes of death (diabetes, cerebrovascular diseases, myocardial infarction Alzheimer’s disease Parkinson’s diseases) associated with PFAS exposure.
Simpson et al., 2013 (10)	Cohort longitudinal	Participants from —the community-based 2005–2006 C8 Health Project (11) and the cohort of workers in polymer production plant (DuPont Washington Works) (18)	32254	Mean – SD - median **PFOA:** non worker: 70.9 -151.2 - 24.2, workers 324.6 - 920.6 - 112.7, combined cohort: 86.6 - 278.9 - 26.1	Over 50 years	Modest evidence of an association between PFOA and stroke incidence.
Hutcheson et al., 2020 (19)	Cohort	US adults ≧̸20 years old from “The C8 Health Project” (11)	3921 with diabetes 44,285 without diabetes	Median (IQR), natural logarithm **PFHxS**: D 2.8 (1.8-4.3), ND 3.0 (1.9-4.8), **PFOA**: D 28.7(12.9-73.6), ND 27.6 (13.4-70.4), **PFOS**: D 21.4(13.8-31.9), ND 20.1(13.5-29.0) **PFNA**: D 1.3(1.0-1.8), ND 1.4(1.0-1.08)	Approximately 20 years	Serum levels of PFHs and PFOS were inversely associated with stroke in adult with diabetes. PFAS compounds do not increase risk of stroke among persons with or without diabetes.
Lind et al., 2018 (20)	Cohort	Adults ≧̸70 years old from the “Prospective Investigation of the Vasculature in Uppsala Seniors (PIVUS) study”.	602	Median and interquartile range **PFHpA**:0.05 (0.03-0.09), **PFHxS**: 2.08 (1.6-3.42), **PFOS**: 13.23 (9.95-17.77), **PFOA**: 3.3 (2.52-4.39), **PFNA**: 0.70 (0.52-0.97), **PFDA**: 0.31 (0.24-0.40), **PFOSA**: 0.11 (0.07-0.17), **PFUnDA**: 0.28 (0.22-0.37)	Follow-up 10-year	PFASs were related to the increase in carotid intima-media thickness. PFNA, PFDA, and PFUA, were all related to intima-media thickness in women, while the relationship was negative in men. The plasma concentration of PFUA was significantly related to carotid plaque in women, but not in men.
Lin et al., 2013 (21)	Cohort	Subject 12–30 years old from the “Young Taiwanese Cohort Study”	664	Median (range) **PFOA**:3.49 (0.75–52.2), **PFOS**: 8.65 (0.11–85.90), **PFNA**: 0.38 (0.38–25.4), **PFUA**: 6.59 (1.50–105.7)	Approximately 8 years	Higher serum concentrations of PFOS were associated with an increase of carotid intima–media thickness.
Mobacke et al., 2018 (22)	Cohort	Subjects ≧̸70 years old from the PIVUS study	801	Mean ± SD **PFHpA**: 0.7 ± 0.7, **PFHxS**: 3.41 ± 3.64, **PFOS**: 14.9 ± 8.88, **PFOA**: 3.59 ± 1.69, **PFNA**: 0.8 ± 0.43, **PFDA**: 0.34 ± 0.15, **PFOSA**: 0.14 ± 0.14, **PFUnDA**: 0.31 ± 0.14.	Follow-up 10-years	PFASs were not significantly related to left ventricular mass. PFNA, PFDA and PFUnDA were related to relative wall thickness in a negative fashion. PFNA was also positively related to left ventricular end-diastolic volume.
Mattsson et al., 2015 (23)	Cohort longitudinal	Male Sweden farmers, born during the period 1930–1949	253 with and 253 without a cardio vascular disease	Medians (interquartile range) in person with (CHD) and without cardio vascular disease (non CHD) **PFOS:** CHD 22.8 (10.0), non CHD 22.0 (10.1), **PFOA:** CHD 4.2 (1.8), non CHD 4.0 (2.0), **PFNA:** CHD 0.5 (0.3), non CHD 0.5 (0.4), **PFDA:** 0.2 (0.1), non CHD 0.2 (0.1), **PFHpA:** CHD 0.06 (0.05), non CHD 0.04 (0.04), **PFHxS:** CHD1.6 (0.7), non CHD 1.6 (0.7), **PFUnDA:** CHD 0.2 (0.1), non CHD 0.2 (0.1), **PFDoDA:** CHD 0.02 (0.02), non CHD 0.02 (0.02)	Approximately 14 years	No statistically significant associations between PFASs levels and risk for developing coronary heart disease. A significant association between higher Levels of PFHpA and coronary heart disease.

**EPAH**, 2-(N-ethyl-perfluorooctane sulfona- mido) acetate; **MPAH**, 2-(N-methyl-perfluorooctane sulfonamido) acetate; **PFAS**, Polyfluoro- and perfluoro–alkyl substances; **PFBA**, perfluorobutyric acid; **PFBS**, perfluorobutane sulfonate; **PFDA**, perfluorodecanoic acid; **PFDO**, perfluorododecanoic acid; **PFHP/PFHpA**, perfluoroheptanoic acid; **PFHS,** perfluorohexane sulfonate; **PFHxA**, perfluorohexanoic acid; **PFHxS**, perfluorohexane sulfonic acid; **PFNA**, perfluorononanoic acid; **PFOA**, perfluorooctanoic acid; **PFOS**, perfluorooctane sulfonic acid; **PFPeA**, perfluoropentanoic acid; **PFSA**, perfluorooctane sulfonamide; **PFUnA/PFUA/PFUnDA**, perfluoroundecanoic acid.

Most of the information concerning the incidence of major cardiovascular events or death in the populations exposed to PFAS derives from studies performed in the USA. By examining 1,216 subjects from the 1999 to 2003 NHANES, Shankar et al. found that circulating level of PFOA was positively associated with the presence of self-reported cardiovascular diseases (either coronary heart disease or stroke) and peripheral artery disease (as assessed by measuring the ankle-to-brachial index), independently of confounders including age, sex, ethnicity, smoking habits, body mass index, diabetes mellitus, hypertension and plasma cholesterol (8). In particular, the risk was doubled for cardiovascular disease and 75% higher for peripheral artery disease in subjects grouped in the highest quartile of exposure, as compared to the lowest quartile (16).

In a subsequent NHANES (1999-2014) including 10,859 participants with self-reported cardiovascular events (extending the previous definition by adding congestive heart failure, angina pectoris and heart attack), the association was maintained also considering a broader spectrum of PFAS. Using multivariable-adjusted models, total PFAS concentration was positively associated with total cardiovascular disease (an increase of nearly 45% in odds ratio was observed for the higher quartiles), independently of traditional cardiovascular risk factors. Individual PFAS (PFOS, PFUA, MPAH, EPAH, PFDO, PFSA and PFBS) were associated with total events and individual PFAS showed different positive associations with individual events, such as MPAH and PFDO with congestive heart failure and PFNA, PFDE and PFUA with coronary heart disease (2).

An ecological observational study compared mortality for causes of death selected on the basis of previously reported associations was performed in the Veneto region (Italy) during the period 1980-2013 in municipalities with PFAS-contaminated and PFAS-uncontaminated drinking water. In that study, a statistically significant increase in the rate ratio for mortality related to cardiovascular diseases, including diabetes mellitus (1.21), cerebrovascular diseases (1.34) and, mainly, myocardial infarction (1.22) was observed in the PFAS contaminated municipalities compared to PFAS-uncontaminated municipalities (17).

A large community survey called the C8 Health Project involved thousands of people living in one of the six districts with PFAS-contaminated water in West Virginia and Ohio (11). In that survey, 1,596 self-reported cases of stroke were assigned, of whom 919 were validated by medical records. In the retrospective analysis, higher quintiles of cumulative exposure had hazard ratios that spanned between 1.13 (statistically non-significant) to 1.45 (significant), compared to the lowest quintile, without a linear trend. In a prospective analysis, no statistically significant relationships were found. The conclusion of the authors was that the study provided only limited evidence of an association between PFOA and stroke incidence (10).

In a more recent analysis of the C8 Health Project, data were stratified according to the diagnosis of diabetes mellitus: 238 cases of stroke occurred in a total of 3,921 adults with diabetes and 643 cases occurred among 44,285 adults without diabetes. Statistically significant inverse association of stroke with serum PFHxS and PFOS, two compounds not investigated in the previous survey, was observed only considering subjects with diabetes mellitus, while no association was detected in patients without diabetes and exposure to other PFAS (19). According to the authors, a survival bias may exist in that participants with particularly high levels of PFAS in the blood could have died before the beginning of the study.

Exposure to PFAS may increase the risk of arterial thromboembolism by promoting atherosclerosis-related vascular disease. To this aim, a number of studies examined early and advanced signs of atherosclerosis in exposed populations, particularly in young people, in whom it was easier to discriminate the role of exposure to PFAS from confounding conditions (**Table 1**).

Relation between either carotid artery or cardiac remodelling and PFAS have been identified in exposed subjects in cross-sectional and longitudinal studies. In the Prospective Investigation of the Vasculature in Uppsala Seniors (PIVUS) study, a total of 602 participants were investigated every 5-years starting at 70 years of age. The baseline concentrations of 6 different PFAS were significantly associated with the increase in carotid intima-media thickness over a 10-year follow-up, even after adjustment for traditional cardiovascular risk factors (20).

In a cross-sectional study, highly significant interactions were observed between some PFAS and sex regarding both intima-media thickness and the prevalence of atherosclerotic plaques. PFNA, PFDA, and PFUA, were all related to intima-media thickness in women, while the relationship was negative in men. The plasma concentration of PFUA was significantly related to carotid plaque in women (odds ratio 1.59), but not in men (odds ratio 0.93) (20).

From a population-based sample (the Young Taiwanese Cohort Study), 664 adolescents and young men were selected for a study that evaluated the relation between PFAS and subclinical atherosclerosis. After controlling for covariates, including traditional cardiovascular risk factors, PFOS was significantly associated with an increase in carotid intima-media thickness, especially in female adolescents, non-smokers, non-overweight and carriers of apolipoprotein E genotype allele E2 and E3/E3, which play a key role in lipid transport and metabolism and are important genetic markers for hyperlipidaemia, coronary heart disease and ischemic stroke (21).

In the already mentioned PIVUS study, 801 subjects aged 70 years or more were investigated in a cross-sectional study also for left ventricular geometry, as determined by echocardiography. None of the eight evaluated PFAS were significantly related to left ventricular mass. After correction for traditional cardiovascular risk factors, PFNA, PFDA, and PFUA were associated to relative wall thickness in a negative manner. Besides being inversely related to relative wall thickness, PFNA was also positively associated with left ventricular end-diastolic volume, suggesting a role for PFAS in cardiac remodelling (22).

The studies so far completed show, however, conflicting results concerning exposure to PFAS and specific cardiovascular events. In fact, Hutcheson et al. reported no relation in the whole studied population of 44,285 exposed subjects or inverse relation in a subgroup of diabetic patients and the risk of stroke from the C8 health study (19). Similarly, no clear relation was found between PFAS exposure and the risk of coronary artery disease in a longitudinal case-control study involving male farmers from nine different rural districts in Sweden born during the period 1930–1949 (23).

Differences in total and maximum exposure might explain the differences in the results concerning exposure to PFAS, cardiovascular events and target organ damage. **Table 1** offers a summary of the discussed studies.

## PFAS-Related Metabolic and Hypertensive Disorders

Several studies examined the relationship between exposure to PFAS and the metabolic alterations that may be determinants of thrombotic cardiovascular disease, promoting atherosclerosis. Being endocrine disruptors, PFAS have been extensively investigated for their potential effects on lipid and glucose metabolisms. The effects of PFAS on blood pressure and the development of arterial hypertension have also been recently investigated. **Table 2** summarizes the main studies concerning exposure to PFAS and metabolic alterations, some of which are discussed below.

**Table 2:** Longitudinal studies exploring the association between PFAS and glucose metabolism/diabetes mellitus.

Author, year and reference	Study Design	Population	n. of subjects	PFAS plasma concentration (ng/mL)	Observation period	Main Results
Leonard RC, 2008 (18).	Retrospective cohort	Workers in polymer production plant (DuPont Washington Works)	6027	n.a.	Mean (SD): Males 26 (15); F 16 (10) years	Mortality associated with diabetes significantly increases in workers in polymer production plant.
Lundin JI, 2009 (24).	longitudinal	Workers in polymer production plant (3M Company plant, Minnesota)	3993	n.a.	Mean 31.3 years	A work history of only moderate-exposure was associated with the risk of dying from diabetes mellitus.
Steenland K, 2015 (25).	Cohort	Workers in polymer production plant (DuPont Washington Works)	3713	Median (SD) **PFOA**: 325 (920)	10 years	Very modest positive trend using the categorical trend test for T2DM.
Domazet SL, 2016 (26).	Cohort	Danish children (European Youth Health Study)	201/202	Median (IQR) **PFOS** M 44.5 (35.4-55.7); F 39.9 (34.3-49.3); **PFOA** M 9.70 (7.7-12.1), F 9.0 (7.4-11.2)	6-12 years	PFOA exposure in childhood was associated with decreased β-cell function at 15 years of age.
Fleisch AF, 2017 (27).	Cohort	US children (Project Viva)	665	Range in the 4th quartile **PFOA** (8.0–22.4); **PFOS** (34.9–168.0); **PFNA** (1.0–2.6); **PFHxS** (3.8–43.2)	Median 7.7 years	Children with higher PFAS concentrations had lower HOMA-IR, especially females.
Matilla-Santander N, 2017 (28).	Cohort	Spanish pregnant women (INMA study)	1204	Range in the 4th quartile **PFOS** Q4: (7.81-38.58); **PFOA:** (3.30-31.64); **PFHxS:** (0.82-11.00); **PFNA:** (0.90-5.51)	From the 1^st^ trimester to delivery	Serum PFOS/PFHxS was associated with impaired glucose tolerance and with GDM.
Jensen RC, 2018 (29).	Cohort	Danish pregnant women (Odense Child Cohort)	318	Median (5th–95th percentile) **PFOS**: 8.31 (4.08–16.26); **PFOA**: 1.71 (0.69–4.19); **PFHxS:** 0.30 (0.08–0.60); **PFNA**: 0.66 (0.37–1.58); **PFDA:** 0.26 (0.15–0.53)	Between the 11^th^ and 28^th^ gestational weeks	In women with high risk for GDM, a two-fold increase in PFHxS concentration was associated with increased FBG, fasting INSULIN and HOMA-IR; a doubling in PFNA concentration was associated with higher fasting insulin and HOMA-%β.
Mancini FR, 2018 (30).	Cohort	French women (E3n Cohort Study)	71270	Estimated mean dietary exposure to **PFOS** 0.49 (0.18) ng/kg body weight/day; **PFOA** 0.86 (0.73) ng/kg body weight/day	Over 15 years of follow-up	Inverse U-shape association was found when considering PFOA and T2DM; PFOS was nonlinearly associated with T2DM only in women with BMI ≤ 25 kg/m^2^.
Wang H, 2018 (31).	Cohort	Chinese pregnant women (Tangshan City)	560	Range min-max **PFOS: (**0.8-114.6); **PFOA: (**1.2-77.7)	From the 1^st^ trimester to OGTT (mid-pregnancy)	PFOA was positively associated with HOMA-IR and blood glucose level at 1 h and 2 h of OGTT; PFOS tended to be negatively associated with FBG and OGTT blood glucose.
Alderete TL, 2019 (32).	Cohort	Overweight Hispanic children (8-14 ys) included in the SOLAR project	40	Geometric mean (SD) **PFHxS:** 1.65 (2); **PFOS:** 12.22 (1.91); **PFOA**: 2.78 (1.29)	1-3 years	The increase in PFOA and PFHxS concentrations was associated with an increase in 2-hour glucose levels. The increase in PFHxS concentrations was also associated with an increase in the glucose area under the curve.
Rahman ML, 2019 (33).	Cohort	Pregnant women (NICHD Fetal Growth Study)	2334	Geometric mean (95%CI) **PFOS**: 5.21 (5.07-5-35); **PFOA:** 1.99 (1.93-2.04); **PFHxS:** 0.76 (0.73-0.78); **PFNA:** 0.80 (0.78-0.82); **PFDA:** 0.27 (0.26-0.28)	Between 8-13 weeks to delivery	PFNA, PFOA, PFHpA, PFDoDA showed significant positive associations with GDM among women with a family history of T2DM.
Zhang C, 2015 (34).	Prospective case-control	Pregnant women (LIFE study)	258	Geometric mean (range) **PFOA** non-GDM: 3.07 (2.83–3.12); **PFOA** GDM: 3.94 (3.15–4.93)	From pre-conception to >24 weeks of gestation	Positive association between serum PFOA and GDM.
Sun Q, 2018 (35).	Prospective case-control study	US women (Nurses’ Health Study II)	793 cases and 793 controls	Mean (IQR) in cases **PFOS:** 35.7 (26.4–48.3); **PFOA**: 4.96 (3.70–6.67)	Follow-up: 6:7 ± 3:7 y	Higher plasma concentrations of PFOS and PFOA were associated with an elevated risk of T2DM.
Liu X, 2019 (36).	Prospective nested case-control	Chinese pregnant women (Beijing)	439	Median (IQR) long chain **PFOS**: 4.16 (2.79–6.39); long chain **PFOA** 2.29 (1.78–3.12)	From the 1^st^ prenatal care visit to 24-28 gestational weeks	Short-chain PFCAs exposure and both GDM risk and impaired glucose homeostasis in pregnant women.
Yu G, 2021 (37).	Cohort	Pregnant women who participated in the Shanghai Birth Cohort	2747	Median (IQR): **PFOA:** 11.55 (5.93); **PFOS:** 9.40 (6.90); **PFNA:** 1.65 (1.07)	24 - 28 weeks	Environmental exposure to PFAS may affect glucose homeostasis in pregnancy and increase the risk of GDM, especially in normal weight women.
Valvi D, 2021 (38).	Cohort	Farohese Islan born individuals	699	Median (min-max): **PFOS** 31.5 (7.23-106.1); **PFOA** 5.06 (1.31-17.3); **PFHxS** 0.92 (0.19-55.2); **PFNA** 0.69 (0.12-2.88)	28 years	Associations were stronger for PFOS and suggested decreased insulin sensitivity and increased β-cell function.
Charles D, 2020 (39).	Nested case-control study	Norwegan Women and Cancer Study	46 T2DM vs. 85 non- T2DM	Median (5th–95th percentile) in cases: **PFOA**: 2.32 (1.00, 4.13); **PFOS:** 20.1 (10.3, 37.9); **PFNA:** 0.38 (0.18, 1.06)	Nearly 4 years	No significant associations between pre-diagnostic PFAS concentrations and T2DM incidence.
Girardi P, 2021 (40).	longitudinal case-control	Male employees for a factory that produced PFOA and PFOS	462	Geometric Mean (min-max): **PFOA:** 4048 ng/mL (19–91,900)	31.7 years	Increased relative risk for mortality from diabetes consequences in the cohort of workers for a factory that produced PFOA and PFOS as compared to the cohort of workers from the metalworking factory.
Cardenas A, 2019 (41).	Cohort from a randomized controlled study	Participants from the Diabetes Prevention Program (DPP) trial and Diabetes Prevention Program Outcomes Study (DPPOS)	957	Geometric Mean (IQR): **PFOA** 4.82 (3.20); **PFOS** 18.42 (16.90); **PFHxS**: 2.41 (2.40); **PFNA**: 0.53 (0.40)	15 years	A doubling in baseline branched PFOA concentration was associated with a 14% increase in diabetes risk for the placebo but not in the lifestyle intervention group.

BMI, Body Mass Index; FBG, Fasting Blood Glucose; GDM, Gestational Diabetes Mellitus; HOMA-IR, Homeostatic Model Assessment for Insulin Resistance; HOMA-%β, Homeostatic Model Assessment for β-cell function; OGTT, Oral Glucose Tolerance Test; T2DM, Type 2 diabetes mellitus. **PFDA,** perfluorodecanoic acid; **PFHxS,** perfluorohexane sulfonic acid; **PFNA**, perfluorononanoic acid; **PFOA**, perfluorooctanoic acid; **PFOS**, perfluorooctane sulfonic acid.

In 888 pre-diabetic adults from the Diabetes Prevention Program (DPP) and DPP Outcomes Study with PFAS plasma concentrations available at baseline (1996-1999), plasma lipid concentration was repeatedly assessed over 15 years of follow-up. An association between some PFAS, including PFOA, PFHxS, and PFNA, and total cholesterol was observed both at baseline and longitudinally. Interestingly, this association was statistically significant only in patients who did not receive lifestyle intervention, suggesting that active intervention can mitigate the deleterious effect of these organic chemicals on lipid metabolism (42).

A large population-based cross-sectional observational study performed in the Veneto region found an association between some PFAS (in particular PFOA, PFOS, and PFHxS) and total, HDL and LDL cholesterol, as well as between PFOA,PFHxS and triglycerides (43, 44).

Positive association between PFAS exposure (predominantly to PFOS and PFHxS) and serum lipids was also found in a Swedish adult population (1945 adults aged 20–60), especially for total cholesterol and LDL cholesterol (45).

Using cross-sectional data from 7,904 adults in the 2003-2012 NHANES, a strong positive association was found between serum PFOA and diabetes prevalence in men with an adjusted model (odds ratio 2.66), but not in women (odds ratio 1.47, non-significant) (46).

Other studies did not find a similar association or even found an inverse association. Conway and colleagues, examining 6,460 individuals with and 60,439 without diabetes mellitus from the C8 Health Project, found that PFAS levels were significantly lower in those with type 1 and type 2 diabetes, with similar results for PFHxS, PFOA, PFOS and PFNA (47, 48). In a small study including 122 men in Hvar (Croatia), only PFNA, but not PFOS and PFOA, were significantly associated with increased risk of metabolic syndrome (49).

In 1871 adults from the NHANES 2013-2014, high plasma PFOA was associated with an increase in total cholesterol and enhancement in β-cell function, as estimated by the updated Homeostasis Model Assessment (HOMA2) index (50), whereas branched PFOA was significantly associated with increased fasting glucose. All PFOA isomers positively associated with HDL cholesterol and negatively associated with glycated haemoglobin. Branched PFOS positively associated with β-cell function (50). As for plasma glucose, insulin secretion, pancreatic β-cell function and insulin resistance in children and adolescents the results are mixed with some studies suggesting a deleterious effect of PFAS (51, 52) and some others either no effect (53, 54) or an inverse association (27, 55). Therefore, no firm conclusion can be drawn on the effect of PFAS in diabetic patients. This could be at least partly due to different exposure in the so far available studies. A brief overview of the longitudinal studies evaluating the link between PFAS exposure and either diabetes incidence or the alteration of glucose homeostasis is presented in **Table 2**. In summary, longitudinal studies with an adequate follow-up and with the highest exposure to PFAS, witnessed by plasma level of these compounds, show that at least some PFAS are associated with elevated plasma lipids and tend to impair glucose tolerance, especially *via* an increase of insulin resistance. Interestingly, the same is true even during pregnancy, a unique situation of physiological insulin resistance. The relationship between body weight and exposure to PFAS was specifically examined in some observational studies. In a prospective cohort study, data from 957 participants of the Diabetes Prevention Program trial, conducted from July 1996 to May 2001, and the Diabetes Prevention Program Outcomes Study, performed from September 2002 to January 2014, were analysed. Each doubling in total PFAS concentration was associated with a 1.80 kg increase in body weight from baseline to 9 years after randomization in the placebo group, but not the lifestyle intervention group. Similarly, each doubling in PFAS was associated with a 1.03-cm increase in hip girth in the placebo, but not the lifestyle intervention group. No associations were observed for changes in mean waist circumference (56).

In the 2-year POUNDS LOST randomised clinical trial, baseline concentrations of major PFAS were measured in 621 overweight and obese participants aged 30-70 years. Bodyweight and resting metabolic rate were measured at baseline and 6, 12, 18, and 24 months after. Higher baseline levels of PFAS were significantly associated with a greater weight regain. Higher baseline plasma PFAS concentrations, especially PFOS and PFNA, were significantly associated with a greater decline in resting metabolic rate during the weight-loss period and less increase in resting metabolic rate during the weight regain period in both men and women (57).

In the already cited study by Yang et al., circulating PFHxS, PFOA and PFNA were significantly higher not only in subjects with metabolic syndrome but also in obese subjects (58).

However, the already cited study in older male anglers in Wisconsin who typically had high dietary fish consumption, increasing body mass index was associated with lower PFAS levels (59).

In a small observational study from Physical Examination Center affiliated to Capital Medical University, China, 148 male subjects including 81 affected by metabolic syndrome and 67 non-affected by metabolic syndrome as reference were recruited. By logistic regression analysis, the plasma concentration of PFNA was associated with a 10.9 fold increase in the risk of metabolic syndrome. Moreover, increased serum PFNA concentrations were associated with high systolic and diastolic blood pressure (odds ratio 7.52 and 7.27 respectively), hypertriglyceridemia (odds ratio 13.2) and obesity (odds ratio 13.3) (58).

As for the studies about obesity, when the longitudinal ones are selected, the effect of PFAS on the tendency on weight gain is more evident, even if factors related to lifestyle can alter the association. Concerning the relationship between PFAS and blood pressure, a large population-based cross-sectional observational study performed in the Veneto region focused on exposure to PFOA, PFBS, PFOS and PFHxS among highly exposed young adults. The results did not support the hypothesis of an association between PFAS and metabolic syndrome. However, a statistically significant positive association between PFAS plasma concentration and individual components of the metabolic syndrome, namely increased blood pressure and elevated plasma triglycerides, was observed (44).

In 2,934 adults included in two surveys of the NHANES (2003-2004 and 2005-2006), PFOA concentration was associated with both systolic blood pressure and the prevalence of arterial hypertension (the odds ratio more than doubled in participants in the 5th quintile versus the first quintile of exposure) (60). More recently, the cross-sectional analysis of data from 6,967 adults included in the 2003-2012 NHANES confirmed the association of PFOA and PFNA with the prevalence of hypertension, but a J-shaped relationship was detected (61).

In 1,612 Chinese adults aged 22-96 years from Shenyang, increased serum concentration of PFAS was associated with a higher prevalence of hypertension and increased systolic and diastolic blood pressure. Interestingly, females exhibited stronger effects than males and branched PFAS were more consistently associated with blood pressure with respect to linear PFAS. The results showed that increased serum concentrations of all (both branched and linear) PFAS were associated with a higher prevalence of hypertension. Adjusted odds ratios for hypertension per ng/mL increase in PFAS ranged from 1.10 for perfluorobutanoic acid (PFBA) to 1.26 for PFOS, and the estimated increases in mean systolic and diastolic blood pressure ranged from 0.80 mmHg for PFBA to 4.51 mmHg for PFOS and from 0.51 mmHg for PFHxS to 2.48 mmHg for PFNA, respectively (62).

In a small study in Chinese men (81 affected and 67 non-affected by metabolic syndrome), circulating levels of PFOA and PFNA were significantly higher in the subjects with metabolic syndrome, and were also associated with blood pressure (58).Recently, Pitter and colleagues used data from the Regional health surveillance program in the Veneto region (Italy), including 15,786 individuals aged 20–39 years, and found that circulating PFAS concentration was associated with an increase in both systolic and diastolic blood pressure and with the prevalence of hypertension, the latter only in men. In particular, the difference in systolic and diastolic blood pressure from the lowest to highest decile of circulating PFOA was nearly 1.5 mmHg (63).

In more than two-thousand children examined in the NHANES 2003-2012, PFAS level in blood was associated with higher diastolic blood pressure in a linear regression model (64).

The relation between alternative and legacy PFAS and blood pressure was examined in 1,273 healthy Chinese, from the “Isomers of C8 health project”. An increase in circulating PFAS was associated with higher diastolic blood pressure (1.31 mmHg) and an increased prevalence of hypertension. When stratified by sex, the effects of alternative PFAS on blood pressure and hypertension were stronger in women (65).

Also for the association of PFAS with either hypertension or blood pressure some inconsistency exists, since other studies reported either no or negative association.

In a large longitudinal among workers at a Mid-Ohio Valley chemical plant exposed to PFOA (n=3,713) and residents of the surrounding community (n=28,541), cumulative PFOA exposure (sum of yearly serum concentration estimates) was not associated with the incidence of either hypertension or coronary artery disease, whereas was non-linearly associated with the incidence of hypercholesterolemia, especially among males aged 40-60 years (66).

The already mentioned small study on 154 male anglers older than 50 years living in Wisconsin PFAS exposure was associated with a lower risk of hypertension, although the only significant odds ratio was with PFNA (59).

In 171 control subjects from a population-based nested case-control study on type 2 diabetes mellitus, including middle-aged women and men, who were longitudinally followed for several years, association with incidence of hypertension was inconsistent throughout the period (67).

In the Västerbotten Intervention Programme, a sub-cohort in the Northern Sweden Health and Disease Study, repeated measurements of several PFAS were performed and possible association with hypertension and plasma lipids was analysed: only triglycerides were inversely associated with PFOA and PFOS (67).

Thus, regarding the effect of different PFAS on blood pressure, cross-sectional studies show an association of PFAS either with systolic or diastolic blood pressure or the prevalence of hypertension. In contrast, longitudinal studies did not confirm an apparent effect of the exposure on the incidence of arterial hypertension. This fact is not unexpected, due to the multifactorial nature of hypertension. Moreover, in cross-sectional studies, only a few mmHg of increase in blood pressure were detected in people with the highest exposure to PFAS.

## Putative Mechanisms Linking PFAS Exposure to Cardiovascular Damage and Clinical Events: Platelet Activation and Experimental Thrombus Formation

In spite of consistency in epidemiological data, limited information exists concerning the mechanisms underlying the increased risk of cardiovascular events observed. Apart from the metabolic disorder attributable to PFAS exposure, increased oxidative stress, as evaluated *in vivo* using biomarkers or testing *in vitro* the bioactivity of individual PFAS, has been advocated by some authors as a plausible mechanistic link between epidemiologic data and evidence of organ damage (15). Recent experimental studies indicating that PFAS accumulate in cell membranes of cells allowed a more in-depth analysis of the mechanisms leading to the clinical consequences of exposure in humans (14, 15).

A line of evidence supports the hypothesis that platelet-dependent thrombus formation might be a determinant of an increased prevalence of cardiovascular events in subjects exposed to PFAS.

Lin et al. analysed in a cohort of 848 adolescents and young adults the relationship between plasma level of PFOS and increased carotid intima-media thickness. Circulating microparticles (presently named large microvesicles in the official nomenclature) derived from the endothelium (CD62E+ and CD31+/CD42a-) and platelets (CD62P+ and CD31+/CD42a+), along with the urine excretion of 8-hydroxydeoxyguanosine, were analysed as biomarkers of endothelial dysfunction, platelet activation, and oxidative stress-derived DNA damage (68). The results showed that endothelial-derived and platelet-derived microvesicles increase significantly across quartiles of PFOS in a multiple linear regression analysis. Furthermore, the increase in circulating microvesicles corresponded to the rise in the odds ratio of a thicker carotid intima-media (greater than 50th percentile) observed with the higher PFOS concentration (odds ratio=2.86) in a logistic regression model.

Circulating platelet- and endothelium-derived microvesicles increase under pathological conditions including, cancer, sepsis, diabetes cardiovascular diseases and acute coronary syndromes (69–72). Microvesicles that are released from activated platelets and dysfunctional endothelial cells carry on their surface most of the proteins and receptors that are expressed on plasma membranes of the parent cells. In particular platelet-derived microvesicles expose on their surface the prothrombinase complex, while tissue factor is exposed on the surface of endothelium-derived microvesicles (72, 73). Furthermore, large platelet-derived microvesicles express receptors, including the fibrinogen receptor, the vonWillebrand Factor receptor GPIb, and P-Selectin that allow the interaction with blood cells and the endothelium (74). Along with cell-surface proteins, the cytosol of microvesicles contains RNA, miRNA and DNA that can be transferred to target cells (75). Therefore, platelet- and endothelial-derived microvesicles are biomarkers and contributors to vascular inflammation and the development of atherothrombosis. Therefore, the observed correlation of circulating microvesicles with early signs of large artery damage in young subjects exposed to PFAS offers clues on the mechanisms by which PFAS may determine cardiovascular events later in life.

A more direct evidence that PFAS can alter the functionality of platelets derives from the study by De Toni et al. investigating which were the possible blood cells target of PFOA accumulation and the effect of exposure to PFOA (76). By using liquid chromatography/mass-mass spectrometry (LC-MS/MS) to analyse the incorporation of PFAS into cell membranes, they demonstrated that platelets are the major target of PFOA accumulation (about 10% of total blood PFOA) which essentially localized to the cell membrane. A simulation comparing normal platelet membranes containing 30% cholesterol and membranes incorporating 10% PFOA and 20% cholesterol, allowed the authors to conclude that a bilayer configuration including PFOA exhibits an estimated energy about 10% higher than the equilibrium energy of reference bilayers. Accumulation of PFOA in platelet membrane may therefore cause a more fluid configuration which can alter cell permeability and ion channel structure and function as well (76).

To assess the biological relevance of this finding, the authors analysed platelet pre-treated with PFOA 400 ng/ml (a concentration that is observed in chronically exposed subjects). When platelets were stimulated with thrombin receptor activator peptide 6 (TRAP-6), a larger increase in cytosolic calcium concentration was observed and this was accompanied by a larger aggregatory response, compared with platelets without PFOA stimulated with TRAP-6 (76). An increase in free cytosolic calcium concentration is a key signal promoting platelet activation, degranulation and aggregation in response to soluble agonists and ligands of platelet adhesion receptors (77). To demonstrate the biological effects of altered calcium signal, platelet degranulation was investigated in resting and stimulated platelets. Under these experimental conditions, in the presence of 400 ng/ml PFOA resting platelets expressed on their surface, the same amount of P-selectin that was observed in TRAP-6-activated platelets without PFOA. P-selectin exerts a crucial role in aggregation and cell-cell communication by interacting with ligands expressed on monocytes and endothelial cells (78).

To offer converging evidence on the role of PFOA as a platelet “activator”, the authors compared the aggregation profile obtained in blood from 48 male subjects chronically exposed to PFOA with that of 30 subjects who had never been exposed to PFOA. Whole blood platelet aggregation showed an increase in platelet responses to different agonists in exposed subjects.

While the described effects of PFOA could be of relevance for cardiovascular risk in subjects living in heavily polluted areas where accumulation was severe (76, 79), very recent data have been obtained concerning the effects of C6O4 (acetic acid, 2,2-difluoro-2-((2,2,4,5-tetrafluoro-5(trifluoromethoxy)-1,3-dioxolan-4-yl)oxy)-, ammonium salt), an alternative PFAS presently used in a variety of industrial applications (80).

The described capacity of PFOA to interact with cell membranes led to explore whether platelets can also adsorb the fluorinated compound C6O4 (76, 79). The hypothesis that C6O4 could be taken up by platelet was investigated analysing the concentration of that compound in isolated and washed platelets after incubation with an exploratory concentration of 500 ng/mL C6O4 (80). A highly significant increase in C6O4 content in platelets (about 30 times compared to the basal levels) was observed. The site of C6O4 accumulation was determined by LC-MS/MS. This analytical process revealed that the majority of C6O4 localised to the plasma membrane fraction and to a lesser extent to the cytosolic fraction, supporting the hypothesis that C6O4 accumulates in human platelets, as previously observed with PFOA.

Based on computational docking analysis, the binding of C6O4 to phosphatidylcholine creates a complex, suggesting a possible interaction of C6O4 with phospholipids that confers to the cell membranes a more fluid or an easily destabilised structure, suggesting a stronger influence of C6O4 exposure on the cell-cell surface interaction (80, 81). Using the bilayer fluidity-sensitive probe Merocyanin 540, a significant and progressive increase in staining intensity was observed after C6O4 incubation at different concentrations. Taking together these data and the results of the docking analysis, a major influence of C6O4-platelet membrane interaction on platelet function could be anticipated.

To test the biological effects of *in vitro* exposure of platelets to C6O4 (100 - 200 ng/mL), adhesion-dependent platelet aggregation under flow was investigated either in presence or absence of acetylsalicylic acid. Using a specific microfluidic biochip pre-coated with collagen and perfused with platelet-rich plasma at a predefined shear rate to mimic *in vivo* thrombus formation, a significant increase of the area covered by platelet aggregates was observed after treatment with C6O4. Incubation with acetylsalicylic acid significantly reduced thrombus formation under flow condition (80).

The consequences of exposure to C6O4 were further investigated *in vitro* by measuring platelet aggregation. A statistically significant increase in platelet response to arachidonic acid was observed with 1, 10, 100 – 500 ng/mL C6O4, with accelerated platelet response and increased maximum platelet aggregation. Interestingly a clear dose-response effect of C6O4 was not observed. Similar results were obtained using ADP or collagen. These aggregatory responses were blunted or almost abolished by coincubation with acetylsalicylic acid. The “activatory” effects of C6O4 on platelets were further explored by measuring the generation *in vitro* of large microvesicles expressing C41 and binding annexin V (maker of a procoagulant activity). Statistically significant increase in procoagulant microvesicles generation was observed when platelets were stimulated with different agonists in the presence of C6O4. All these effects were blunted by acetylsalicylic acid (80). The studies concerning the effects of PFAS on platelets are summarized in **Table 3**.

**Table 3:** Clinical and experimental studies exploring the mechanisms of action of PFAS in platelet and thrombus formation.

Author, year and reference	Study Design	Population and n. of subjects	PFAS plasma concentration/*in vitro* exposure (ng/mL)	Biomarker/Platelet function test	Main Results
Lin CY et al., 2016 (68)	Case-control. *Ex vivo*: analysis of biomarkers	Exposed subjects 331 males and 517 females from the study population of the YOTA cohort 97 subjects in the control group. Collectively 886 subjects were studied.	**PFOA:** 3.21 (3.00–3.46), **PFOS:**, 6.44 (6.05–6.89), **PFNA:** 1.21 (1.12–1.31) **PFUA:** 6.39 (5.99–6.82) mean (95% CI)	Microvesicle release from platelets and endothelial cells.	Endothelial microvesicles and platelet-derived microvesicles increase significantly across quartiles of PFAS exposure. The increase in circulating microvesicles was related to the increase in the odds ratio of thicker carotid intima-media.
De Toni et al., 2020 (76)	Case control. *in vitro* and *ex vivo*: analysis of platelet function	Healthy subjects in the *in vitro* study. Exposed subjects were 48 male subjects living in a specific area of the Veneto region with high PFAS environmental pollution, compared with 30 low-exposure control subjects.	Blood incubated for 30 minutes with 25-1000 ng/mL **PFOA**. Subjects with prolonged exposure to PFOA: plasma level 128.0 ± 48.5, PFOA platelets level: 37.2 ± 15.8. Controls: plasma PFOA 4.7 ± 2.1. PFOA platelet level undetectable. Mean ± SD	Whole-blood platelet aggregation. Platelet secretion. Intracellular signals.	*In vitro* study: Increased aggregation induced by soluble agonists, increased free intracellular calcium and increased expression of P-selectin in PFOA-treated platelets. *Ex vivo* study: Increased platelet aggregation in subjects exposed to PFOA, compared to controls.
Minuz P et al., 2021 (80)	*In vitro*: analysis of platelet function	Healthy subjects	Platelet rich plasma incubated for 30 minutes with 1-500 C6O4	Platelet aggregation Microvesicle release. Thrombus formation under flow.	Increased platelet aggregation and release induced by soluble agonists in platelets treated with C6O4. Acetylsalicylic acid blunted all the tested platelet responses.

**PFNA**, perfluorononanoic acid; **PFOA**, perfluorooctanoic acid; **PFOS**, perfluorooctane sulfonic acid; **PFUA**, perfluoroundecanoic acid.

## General Conclusions

**Figure 1:**
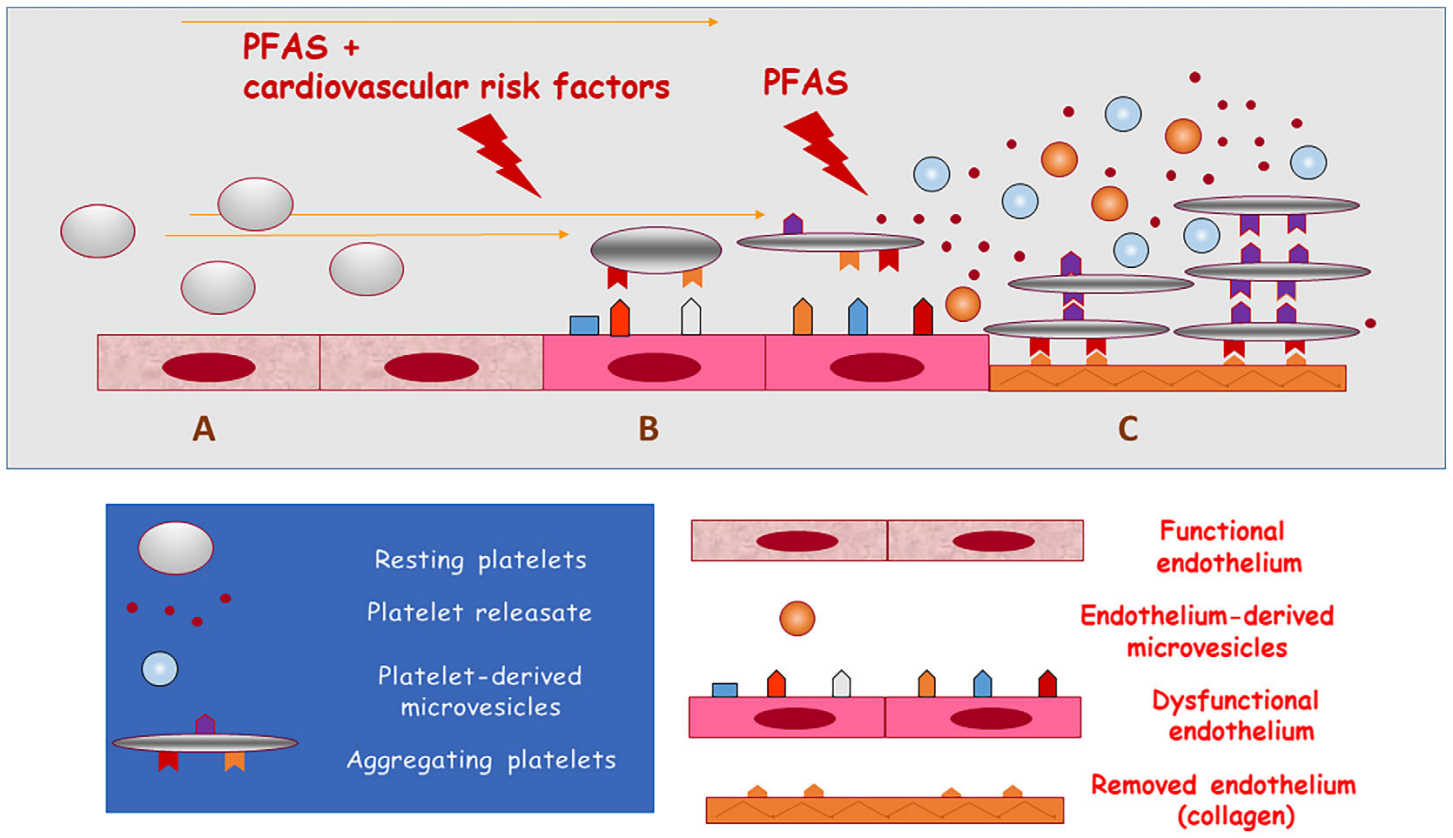
Updated hypothesis on the association of exposure to PFAS with cardiovascular disease and death. PFAS may promote arterial thrombosis by increasing cardiovascular risk (causing lipid and glucose disorders and arterial hypertension) or by directly activating platelets. **(A)** functional endothelial and resting circulating platelets; **(B)** exposure to PFAS and cardiovascular risk factors induce endothelial dysfunction and the activation of circulating platelets (release of microvesicles); **(C)** exposure to PFAS directly induces platelet adhesion and aggregation when endothelium has been removed or damaged and collagen is exposed to blood flow (release of prothrombotic substances and microvesicles from aggregating platelets) thus causing arterial thrombosis.

Cumulating evidence indicates that the cardiovascular system may be the target of PFAS toxicity by increasing the risk of atherosclerosis-related thromboembolic events. This may occur through two converging mechanisms: worsening of cardiovascular risk factors and a direct prothrombotic activity (**Figure 1**). Several studies indicate that metabolic disorders, including hyperlipidaemia, diabetes mellitus, obesity and increased blood pressure may translate into increased cardiovascular events in the exposed populations, possibly through the endocrine disrupting activity of PFAS.

New data from mechanistic studies suggest that platelets may be the target of PFAS, altering the characteristics of plasma membranes, apparently in a dose-independent manner. This may trigger *in vivo* thrombus formation. Notably, all the experiments performed to evaluate the effects of legacy and alternative PFAS on platelets were performed testing *in vitro* concentrations similar to those that have been found in humans chronically exposed to PFAS, or directly examining platelets from exposed subjects. These findings indicate that PFAS accumulate rapidly and promote activation of either unstimulated and stimulated platelets, thus supporting the hypothesis that platelet-centred mechanisms may be implicated in the observed increase in cardiovascular events reported in chronically exposed populations. Large-scale cohort studies using appropriate biomarkers and more data from basic research are required to further validate the hypothesis of a link between PFAS and cardiovascular events and define the strategies to mitigate the cardiovascular risk in exposed populations.

## Author Contributions

All authors listed have made a substantial, direct, and intellectual contribution to the work, and approved it for publication.

## Conflict of Interest

The authors declare that the research was conducted in the absence of any commercial or financial relationships that could be construed as a potential conflict of interest.
